# Risk factors associated with *Campylobacter* detected by PCR in humans and animals in rural Cambodia

**DOI:** 10.1017/S095026881600114X

**Published:** 2016-06-23

**Authors:** K. OSBJER, S. BOQVIST, S. SOKERYA, K. CHHENG, S. SAN, H. DAVUN, H. RAUTELIN, U. MAGNUSSON

**Affiliations:** 1 Division of Reproduction, Department of Clinical Sciences, Swedish University of Agricultural Sciences (SLU), Uppsala, Sweden; 2 Department of Biomedical Sciences and Veterinary Public Health, SLU, Uppsala, Sweden; 3 Centre for Livestock and Agriculture Development, Phnom Penh, Cambodia; 4 National Institute of Public Health, Phnom Penh, Cambodia; 5 National Veterinary Research Institute, Phnom Penh, Cambodia; 6 Department of Medical Sciences, Clinical Microbiology, Uppsala University, Uppsala, Sweden

**Keywords:** Epidemiology, household practice, prevalence, rural household, zoonosis

## Abstract

*Campylobacter* are worldwide-occurring zoonotic bacteria, with the species *Campylobacter jejuni* and *C. coli* commonly associated with diarrhoea in children in low-income countries. In this cross-sectional study, the prevalence of *C. jejuni* and *C. coli* in human and livestock faecal samples was detected by PCR and zoonotic risk factors associated with human *Campylobacter* positivity were identified. In total 681 humans and 753 livestock (chickens, ducks, pigs, cattle) from 269 households were sampled. Children aged <16 years were more frequently *Campylobacter* positive (19%) than adults (8%) and multilevel logistic models revealed that human *C. jejuni* positivity was associated with the following household practices: home-slaughtering [odds ratio (OR) 2·4, *P* = 0·01], allowing animals access to sleeping and food preparation areas (OR 2·8, *P* = 0·02), and eating undercooked meat (OR 6·6, *P* = 0·05), while frequent consumption of beef was protective (OR 0·9, *P* = 0·05). Associations were stronger for home-slaughtering (OR 4·9, *P* = 0·004) with *C. jejuni* infection in children only. *Campylobacter* was highly prevalent in pigs (72%) and chickens (56%) and risk factors associated with human *Campylobacter* positivity were identified throughout the meat production chain. The findings underline the importance of studying source attributions throughout the production chain and the need for upgraded understanding of *Campylobacter* epidemiology in low-income countries.

## INTRODUCTION

Gastroenteritis is a major public health concern, with over 800 000 fatalities in children annually, most occurring in Asia and Africa [1]. Despite a global decline, diarrhoeal mortality accounts for one in ten child deaths in resource-poor countries and gastroenteritis is known to be closely associated with malnutrition and underweight [1, 2]. *Campylobacter*, belonging to the most commonly detected pathogens in children with moderate-to-severe diarrhoea in Asia [3–5], are the most common cause of human bacterial gastroenteritis worldwide [6, 7]. In campylobacteriosis symptoms range from acute abdominal pain, diarrhoea and fever to late sequelae such as reactive arthritis and, although rarely occurring neurological Guillain–Barré syndrome [8]. Of all *Campylobacter* species, *C. jejuni* and *C. coli* are the most common causes of human infection [9].

The epidemiology of human campylobacteriosis appears to differ between high- and low-income countries [10, 11]. In high-income countries symptomatic infection occurs in all age groups [8], whereas in low-income countries most symptomatic *Campylobacter* infections are diagnosed in young children and adults seem to acquire a level of protective immunity following repeated exposure [10, 11]. The global distribution of *Campylobacter* is attributed to asymptomatic colonization of the intestinal tract in a wide range of livestock species [9]. Zoonotic transmission to humans is significant and source-attribution studies in high-income countries have recognized direct contact with farm animals and consumption of chicken, unpasteurized dairy products and contaminated water as being important [12, 13]. International travel, particularly to tropical regions, has however, been suggested as the most important risk factor in high-income countries, involving practices during travel such as eating vegetable salad and raw or undercooked pork [11, 14]. In low-income countries, sources have been less well examined. For rural households in Egypt, the presence of poultry manure, uncovered litter in house yards and lack of barriers to keep animals out of houses have been identified as risk factors for *Campylobacter* infection in children [15, 16], while a study carried out in Ethiopia identified exposure to domestic animals as a sufficient risk factor for infection [17].

In Cambodia, 80% of the population live in rural areas and smallholder farmers represent the majority of livestock producers [18, 19]. Livestock are predominantly reared in free-range systems, with close interaction between livestock and humans and thus enabling exposure to zoonotic pathogens [18]. In such rural and often resource-scarce households, the burden of malnutrition and diarrhoeal disease is high, particularly in children aged <5 years [1, 20, 21]. Nonetheless, data on enteropathogens and their source attribution is limited and the role of zoonotic transmission poorly understood. Few studies have focused on detection of enteropathogens in livestock, although *Campylobacter* have been detected by culture in 81% of the poultry carcases available on sale in Cambodian wet markets [22]. Additionally, in a recent study on livestock in neighbouring Vietnam, *Campylobacter* were detected by culture in 32% of poultry and 54% of pigs sampled on low-biosecurity farms [23].

To the best of our knowledge, no previous study has examined factors associated with *Campylobacter* transmission between animals and humans in Cambodian households. The aim of this study was therefore to identify zoonotic risk factors associated with human *Campylobacter* positivity in rural Cambodian households for which the prevalence of *C. jejuni* and *C. coli* in human and livestock faecal samples had been detected by polymerase chain reaction (PCR).

## MATERIAL AND METHODS

### Study design and data collection

**Fig. 1.**
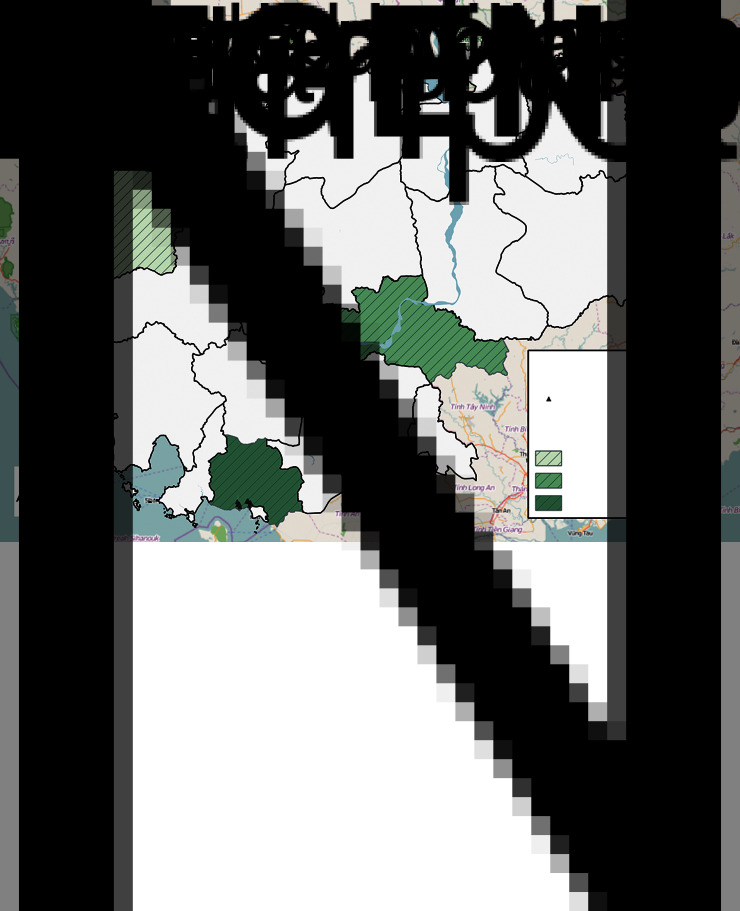
Map of Cambodia showing geographical distribution of the 30 villages included in the cross-sectional study in 2011–2013. Open Development Cambodia (www.opendevelopmentcambodia.net) and OpenStreetMap contributors (openstreetmap.org).

This cross-sectional study was based on our previous studies on household practices [24] and detection of *Campylobacter* by culture and PCR [25] conducted in three regions in Cambodia: Kampong Cham province (in May 2011), Battambang province (in July 2012) and Kampot province (in March 2013) (Fig. 1). In each region, 10 villages were included and in each village, 10 households were selected for interviews and collection of faecal samples. The purposive selection of regions, villages and households has been described previously [24]. The interviews, targeted towards the female head of the household, were carried out in Khmer using a household questionnaire consisting of questions on livestock management, meat consumption and household practices related to zoonosis transmission (Table 1). To enhance consistency between the three regions, the field team was trained in questioning and sampling prior to fieldwork [24]. Each village was visited for two consecutive days. On day 1, selected households were interviewed following consent to participate and provided with containers for human faecal samples. All members of the household were encouraged to provide a faecal sample, regardless of gender, age and history of gastrointestinal symptoms. On day 2, all human samples produced were collected and samples from 1–6 livestock, including chickens, ducks, pigs and cattle (Table 2). Livestock samples were selected depending on the species reared by the household with the aim of covering as many species and age groups as possible. In households farming more than one animal species, a minimum of one sample from each species, was obtained. For each person and livestock sampled, information on age was recorded. In addition, self-reported (or parental report for younger children) gastrointestinal symptoms within a 2-week period prior to sampling were recorded for each sampled person. Geographical position at the central point of the villages included in the study was recorded using a hand-held global positioning system (GPS; Garmin eTrex H).

**Table 1.** Self-reported household practices in the 269 households included in the study (Cambodia, 2011–2013)

What do you practise in this household? (*n* = 269)	No. (%)
Eat undercooked meat	21 (8)
Feed livestock uncooked meat waste	53 (20)
Cull sick animals for consumption	74 (28)
Eat animals found dead	69 (26)
Allow animals access to sleeping and food preparation areas	74 (28)
Slaughter domestic animals	173 (64)
Capture and slaughter wild animals for consumption	18 (7)
Wash hands with soap before and after cooking	240 (89)
Wash hands with soap after handling live animals	229 (85)
Bury or burn meat waste products	218 (81)
Collect manure indoors and outdoors daily	237 (88)

**Table 2.** Number of sampled humans and livestock per household (n = 269) (Cambodia, 2011–2013)

	Number of samples collected per household						
	0	1	2	3	4	5	6
Sample type							
Human	0	55	87	74	37	14	2
Chicken	72	84	90	21	2	0	0
Duck	200	49	18	2	0	0	0
Pig	157	79	29	4	0	0	0
Cattle	138	82	45	4	1	0	0

The target sample size was calculated based on sample size for expected *Campylobacter* prevalence at 95% confidence interval with 5% precision, using the formula presented by Thrusfield [26]. The expected *Campylobacter* prevalence for human samples was set at 20% [27, 28] and the overall prevalence for livestock samples at 35% [29]. An extra 15% was added to the human sample size to adjust for possible confounding and interaction in the statistical models [30]. To account for clustering of infection within households, the target sample sizes were adjusted for intra-cluster correlation, with a coefficient of 0·2 [31]. The average number of humans and livestock sampled per household was set at 3. Thereby an expected *Campylobacter* prevalence of 20% in humans [27, 28] gave a target sample size of 246, which was adjusted to 542 when taking household clustering into account. After adjusting for confounding and interactions, the final target sample size for human samples was set at 623. In livestock, an expected *Campylobacter* prevalence of 35% gave a target sample size of 350, which was adjusted to 840 when accounting for household clustering.

Self-collected human faecal samples were stored on ice-packs until faecal material was transferred by sterile cotton swabs into vials with bacterial freeze medium. Poultry samples were collected by insertion of a swab into the cloaca, while cattle and pig samples were collected by dipping a swab into faecal material collected manually from the rectum. All swabs were placed in vials containing bacterial freeze medium as previously reported [25] and stored in cooler boxes or refrigerated before transportation on ice to Phnom Penh within 1 day for storage at −70 °C pending shipment to Sweden for analysis. Extraction of DNA in livestock samples was carried out at the Swedish University of Agricultural Sciences, and DNA extraction in human samples and all PCR analyses were performed at Uppsala University. Identical multiplex PCR was performed on all human and livestock samples using two specific primers. For *C. jejuni*, the primer pair MDmapA1 upper and MDmapA2 lower targeting the *mapA* gene was used [32], and for *C. coli* we used the primer pair COL3 upper and MDCOL2 lower targeting the *ceuE* gene [32, 33]. A detailed description of the laboratory analyses can be found in Osbjer *et al.* [25].

### Data management and statistical analysis

Data from questionnaires were independently translated by two translators from Khmer into English and compared for consistency before being transcribed into spreadsheets in Microsoft Office Excel 2010 (Microsoft Corp., USA). Statistical analysis was performed in SAS for Windows v. 9.3 (SAS Institute Inc., USA). Statistical tests including Pearson's *χ*^2^, or Fisher's exact test when there were <5 observations per group, were used to analyse differences between age groups in the proportion of *Campylobacter*-positive samples and the proportion of people with gastrointestinal symptoms. An intra-cluster correlation coefficient (ICC) for human and livestock samples detected with *C. jejuni* and *C. coli* was calculated to estimate the correlation between two observations in the same household or village by building unconditional logistic models, extracting the village- and household-level variances and assuming that the person-level variance was 3·29 [34, 35].

To explore potential risk factors for *C. jejuni* and *C. coli* positivity, multilevel logistic models were run with human samples that tested positive for *C. jejuni* or *C. coli* as the outcome variable. Comparable models were also run using the subset: samples positive for *C. jejuni* or *C. coli* in children aged <16 years of age. Univariable models were run for the outcome variables human samples positive for *C. jejuni* or *C. coli* and any of the 11 self-reported household practices (presented in Table 1) as the explanatory variable. Multivariable models were run for the same outcome variables and one of the four groups of explanatory variables: the self-reported gastrointestinal symptoms in sampled humans (presented in Table 3); number of chickens, ducks, pigs and cattle reared in the household; *C. jejuni*- or *C. coli*-positive samples from chickens, ducks, pigs or cattle; and number of days per month that poultry, pork and beef was consumed by the household.

**Table 3.** Rate of self-reported (or parental report for younger children) gastrointestinal symptoms during the 2-week period prior to sampling (n = 681) (Cambodia 2011–2013)

Sample type	Abdominal pain	Diarrhoea	Fever	Vomiting
Child <2 years (*n* = 34)	2 (6)	11 (32)	9 (26)	1 (<1)
Child 2–5 years (*n* = 53)	7 (13)	13 (25)	16 (30)	2 (4)
Child 6–15 years (*n* = 185)	22 (12)	15 (8)	39 (21)	2 (1)
Adult >15 years (*n* = 409)	62 (15)	43 (11)	79 (19)	4 (1)

Values given are *n* (%).

The statistical models had three levels of nested factors in the hierarchy, where each person sampled was clustered within households that were clustered within villages. Variables were considered candidates for multivariable analysis based on their biological plausibility and risk factors previously identified to be associated with human *Campylobacter* infection [12, 15, 16]. Random effects (variables) for households were assumed to be independent and the number of livestock reared and meat consumption were fitted as continuous variables in modelling, with smoothing loess plots applied to assess their functional form [30]. Due to considerable collinearity and interaction, only univariable analysis was performed on the 11 self-reported household practices. The statistical significance level was defined as a two-tailed *P* value ⩽0·05.

QGIS 2·0·1 software was used to map the distribution of villages in open-source base map layers obtained from Open Development Cambodia (www.opendevelopmentcambodia.net) and OpenStreetMap contributors (openstreetmap.org).

### Ethical approval

Ethical approval (43 NECHR, 8 April 2011) was obtained prior to the survey from the National Ethics Committee for Health Research, Ministry of Health, Cambodia, and an advisory ethical statement (Dnr 2011/63) was obtained from the Regional Board for Research Ethics in Uppsala, Sweden. The authors assert that all procedures contributing to this work comply with the ethical standards of the relevant national and institutional committees on human experimentation and with the Helsinki Declaration of 1975, as revised in 2008.

## RESULTS

### Description of included households

A household was defined as a group of people making common arrangements for food and shelter. Interviews and human samples were obtained from 269 households with a median household size of 5·0 people (range 1–17). Of these households, poultry were reared in 253 (94%), pigs in 148 (55%) and cattle in 177 (66%). As described in our previous household study [24], the majority of households reared poultry and cattle in a primarily free-range system, while pigs were reared in a primarily confined system. The mean number of days per month that meat was consumed in the household was 4 for poultry [standard deviation (s.d.) = 4·4], 9 for pork (s.d. = 7·4) and 2 for beef (s.d. = 4·1).

### Self-reported gastrointestinal symptoms in sampled humans

Symptoms of abdominal pain, diarrhoea, fever and vomiting during the 2-week period preceding sampling, as defined by the respondent or for younger children by parental report, were recorded for each person sampled. Fever was the most commonly reported symptom (21%) and showed no statistically significant difference between age groups. However, diarrhoea, was more frequently reported in children aged <6 years than in adults and children aged 6–15 years (*P* = 0·0001) (Table 3).

### Detection of *Campylobacter* in human samples

As reported earlier [25], of the 681 human samples, 82 (12%) tested positive by PCR; *C. jejuni* was detected in 66 samples (80%) and *C. coli* in 16 samples (20%) (Table 4). Children aged <16 years more often tested positive for *C. jejuni* or *C. coli* than adults (*P*<0·001), but no significant difference in the proportion of positive samples could be determined between the three age groups (<2, 2–5, 6–15 years). At least one positive sample was detected in 66 households (24%), with a quite strong clustering of positive samples within households (ICC = 0·14, variance estimate 0·47) and a weaker clustering within villages (ICC = 0·02, variance estimate 0·07).

**Table 4.** Detection of *Campylobacter jejuni* and *C. coli* by multiplex PCR in faecal samples from children and adults in rural Cambodia, 2011–2013

	Number (%) of positive samples			
Method	Child <2 years (*n* = 34)	Child 2–5 years (*n* = 53)	Child 6–15 years (*n* = 185)	Adult >15 years (*n* = 409)
PCR positive for *C. jejuni* or *C. coli*	8 (24)	7 (13)	36 (19)	31 (8)
PCR positive for *C. jejuni*	5 (15)	7 (13)	30 (16)	24 (6)
PCR positive for *C. coli*	3 (9)	0	6 (3)	7 (2)

### Detection of *Campylobacter* in livestock samples

Of the 763 livestock samples obtained from 229 different households, 324 (42%) tested positive for *Campylobacter; C. jejuni* was detected in 165 samples (51%), *C. coli* in 108 samples (33%), and both *C. jejuni* and *C. coli* in 51 samples (16%). *C. jejuni, C. coli* or both were detected in 56% of chickens, 22% of ducks, 72% of pigs and 5% of cattle as presented with stratification by age in Table 5. In the youngest age group of chickens, but not in that of ducks, pigs or cattle, *C. jejuni/C. coli* was more often detected than in the older age groups (*P*<0·001). The number of households with at least one livestock sample analysed by species and the percentage of sampled households with at least one positive sample was 197 (65%) for chicken, 69 for ducks (25%), 112 (78%) for pigs and 132 (6%) for cattle. Clustering of positive samples was weak within households (ICC = 0·05, variance estimate 0·17) and non-detectable within villages.

**Table 5.** Detection of *Campylobacter jejuni* and *C. coli* by multiplex PCR in faecal samples from different age groups of chickens, ducks, pigs and cattle in rural Cambodia, 2011–2013

	Number (%) of positive samples											
	Chicken		Duck			Pig				Cattle		
	<1 yr (*n* = 231)	⩾1 yr (*n* = 104)	<1 yr (*n* = 63)	⩾1 yr (*n* = 28)		<3 mos. (*n* = 59)	3–6 mos. (*n* = 58)	>6 mos. (*n* = 32)		<6 mos. (*n* = 10)	6 mos. to 2 yr (*n* = 33)	>2 yr (*n* = 145)
PCR positive for *C. jejuni, C. coli* or both*	168 (73)	20 (19)	16 (25)	4 (14)		45 (76)	41 (71)	21 (66)		5 (50)	1 (<1)	3 (2)
PCR positive for *C. jejuni*	155 (67)	20 (19)	14 (22)	4 (14)		7 (12)	9 (16)	3 (9)		2 (20)	0	2 (1)
PCR positive for *C. coli*	48 (21)	2 (2)	4 (6)	0		43 (73)	38 (66)	19 (59)		3 (30)	1 (<1)	1 (1)

* Fifty-one samples tested positive for both *C. jejuni* and *C. coli.*

### Analysis of zoonotic risk factors associated with human *Campylobacter* positivity

In the multilevel models, no associations were found between the outcome variables *C. jejuni* or *C. coli* in human samples and self-reported gastrointestinal disease symptoms. Likewise, there were no associations between *C. jejuni* or *C. coli* in human samples and the number of chickens, ducks, pigs or cattle reared or detection of *C. jejuni* or *C. coli* in the household's chickens, ducks, pigs or cattle (Table 6). The household practices of slaughtering domestic animals at home, allowing animals into sleeping and food preparation areas and eating undercooked meat were associated with increased odds of human *C. jejuni* positivity, whereas frequent consumption of beef was associated with decreased odds. The probability of *C. jejuni*-positive samples was higher in the subset models of children aged <16 years for the household practice of home-slaughtering. None of the other household practices listed (Table 1) were associated with *C. jejuni* or *C. coli* in samples from children. Detection of *C. coli* was associated with frequent consumption of poultry, both when all the human samples were included in the model and when the child subset model was used. Frequent consumption of pork was associated with detection of both *C. jejuni* and *C. coli* in the child model (OR 1·1, *P* = 0·04). All models with significant associations between *C. jejuni* or *C. coli* detected in human samples and explanatory variables are presented in Table 6.

**Table 6.** Significant associations in generalized linear mixed models between the outcome variables detection of *Campylobacter jejuni* or *C. coli* by PCR in human samples (n = 681) and samples from children only (n = 272), and explanatory variables measured at the household level (Cambodia, 2011–2013)

Outcome variable	Explanatory variable	OR (95% CI)	*P* value
*C. jejuni* detected in human sample	Slaughter domestic animals	2·4 (1·2–4·8)	0·01
	Allow animals access to sleeping and food preparation areas	2·8 (1·2–6·5)	0·02
	Eat undercooked meat	6·6 (1·0–44)	0·05
	No. of days per month that beef is consumed*	0·9 (0·7–1·0)	0·05
*C. coli* detected in human sample	No. of days per month that poultry is consumed*	1·2 (1·1–1·3)	0·006
*C. jejuni* detected in sample from child aged <16 years	Slaughter domestic animals	4·9 (1·7–14)	0·004
*C. coli* detected in sample from child aged <16 years	No. of days per month that poultry is consumed*	1·2 (1·0–1·4)	0·02

OR, Odds ratio; CI, confidence interval.

* Quantitative explanatory variable.

## DISCUSSION

Our findings suggest that household practices play a role in animal-to-human transmission of *Campylobacter* in rural Cambodian households. The practices of home-slaughtering, allowing animals access to sleeping and food preparation areas, consuming undercooked meat, and frequent consumption of poultry and pork were all associated with an increased probability of human *C. jejuni* or *C. coli* positivity. Children aged <16 years had more than twice the prevalence of *C. jejuni* and *C. coli* found for adults, whereas no difference was identified between older and younger children. Symptoms of diarrhoea were commonly reported, particularly in young children, but gastrointestinal symptoms were not associated with either *C. jejuni* or *C. coli* positivity. Finally, a high prevalence of *C. jejuni* and *C. coli* was detected in poultry and pigs.

In Cambodia, underweight and stunting, markers of acute and chronic malnutrition, are estimated to affect between 28% and 45% of children, with the highest burden seen in rural and resource-poor households [20, 21]. Consumption of a diverse diet, in particular animal-based foods, is a protective factor in malnutrition [21], but poor control of zoonotic pathogens may jeopardize the health benefits. In this study gastrointestinal symptoms were frequently reported in adults and children. Symptoms were self-reported and based on personal perception rather than a set case definition as such method is suggested to reduce recall bias when a recall period of ⩾2 weeks is applied [36, 37]. The absence of associations between *Campylobacter* detection and gastrointestinal symptoms as seen here has been previously reported in low-income countries [38, 39], and is likely due to the development of protective immunity in endemic settings [11]. Frequent exposures to *Campylobacter* at a young age have been shown to boost the immune response with increasing age to protect against clinical disease, but not necessarily against transient positivity [11]. Regardless of symptoms, however, *Campylobacter* positivity is of importance in rural low-income areas, particularly in children, as some studies have also found asymptomatic *Campylobacter* infection to be associated bi-directionally with malnutrition and reduced growth [39, 40]. Some explanations for the absence of association between *Campylobacter* detection and symptoms may also be found in the methods used here. PCR is known to have a high sensitivity in detecting low numbers of live bacteria and also an ability to detect dead bacteria; however, neither of these may be indicative of clinical disease. Detected *Campylobacter* can also reflect convalescent phase as excretion of *Campylobacter* may last up to 10 weeks after infection [11].

In high-income countries the majority of human campylobacteriosis cases seem to be related to chickens [7, 13]. The effect of poultry rearing could, however, not be investigated in this study as nearly all households kept poultry. Livestock keeping *per se* was, in this study, not associated with an increased probability of human *Campylobacter* positivity, even when *Campylobacter* were detected in the livestock reared. Instead, the biosecurity measures and hygiene precautions applied within the household seemed more important. The ICC of 0·14 obtained also shows that human *Campylobacter* infections clustered quite strongly within households, but marginally within villages. The self-reporting used to quantify household practices and disease symptoms has possibly induced some over- and under-reporting resulting from perceived desired responses; however, this approach allowed inclusion of a larger number of households compared to structured observations [41].

Household involvement in slaughtering has not been previously reported as a risk factor for human *Campylobacter* positivity. In this study, the odds for children were higher than for adults, although the actual slaughter was carried out by adults. Possible explanations could be that children are in closer contact with slaughter waste during outdoor play and are less cautious with hand hygiene. Household risk factors associated with human *Campylobacter* positivity were detected throughout the meat production chain here, from free-ranging livestock and home-slaughtering, to unsafe meat preparation and consumption. Such results suggest future actions targeting the entire meat production chain for reduced burden of human *Campylobacter* infection. Moreover, as previously reported, livestock are mainly produced to generate an income and often sold by households [24]. Thus, efficient *Campylobacter* control ought to move beyond the households with improvements in hygiene practices targeting also external factors along the meat production chain, such as middlemen, abattoirs and consumers.

As described by others, associations identified between *Campylobacter* infection and meat consumption are most likely attributable to in-kitchen cross-contamination of food consumed raw, in addition to consumption of meat [42, 43]. Interestingly, in this study, consumption of poultry was associated with human *C. coli,* but not with *C. jejuni* positivity, which is remarkable since *C. jejuni* was detected in 45% of the poultry samples and *C. coli* only in 13%. Nevertheless, some care is needed before generalizing these results, as only 16 human samples tested positive for *C. coli*. Consumption of beef was found protective against human *C. jejuni*, although borderline significantly, but an explanation for this remains unclear. Our data did not support the theory that beef was more frequently consumed in affluent households, affording a higher hygiene standard, or that an increase in beef consumption corresponded to a decrease in poultry and pork consumption (data not shown). Seemingly low odds ratios were obtained for the meat consumption variables due to the unit of 1 day. Odds would increase considerably if meat was consumed 2–3 days extra or more per month. The high odds ratios presented for undercooked meat consumption should, however, be interpreted with caution as the association with *C. jejuni* positivity is borderline significant with a wide confidence interval. The estimated livestock prevalence should also be viewed with caution. Samples were collected at one occasion, thus any intermittent excretion of *Campylobacter* could have been missed. Additionally, the initial 853 livestock samples collected were reduced to 763 after excluding samples from the 31 households where no human samples were obtained, therefore the target sample size of 840 livestock samples was not met.

Surprisingly, and unlike in other studies [10, 16], no differences were identified in *Campylobacter* detection between different age groups of children. One possible explanation could be the previously discussed high sensitivity of PCR detecting low numbers of *Campylobacter* in comparison with culture. Moreover, as previously reported [25], negligible differences were found in *Campylobacter* prevalence between the three regions, which were sampled in different seasons. Seasonal differences are therefore unlikely to have biased the results presented. However, the purposive sampling process in the study, including selection of households with many different livestock species, may have introduced some bias. However, given the high number of households and samples we assume that selection bias had only minor impact on the results and that our sample can serve as an approximation of a population-based design for species-diverse households.

## CONCLUSIONS

Consumption of animal-based foods is important in reducing malnutrition in resource-poor households, but is hampered by the presence of zoonotic pathogens. In this study, *C. jejuni and C. coli* were frequently detected in humans, especially children, and in livestock, especially in pigs and chickens. Several self-reported household practices along the meat production chain from rearing of live animals to meat consumption were found to be associated with *Campylobacter* positivity in humans. These findings underline the importance of studying source attributions of zoonotic enteropathogens throughout the production chain. Finally, an upgraded understanding of the *Campylobacter* epidemiology in low-income countries may guide future interventions aimed at food and nutrition security.

## References

[1] Liu L, Global regional and national causes of child mortality: an updated systematic analysis for 2010 with time trends since 2000. Lancet 2012; 379: 2151–2161. 22579125 10.1016/S0140-6736(12)60560-1

[2] Checkley W, Multi-country analysis of the effects of diarrhoea on childhood stunting. International Journal of Epidemiology 2008; 37: 816–830. 18567626 10.1093/ije/dyn099 PMC2734063

[3] Kotloff KL, Burden and aetiology of diarrhoeal disease in infants and young children in developing countries (the Global Enteric Multicenter Study, GEMS): a prospective, case-control study. Lancet 2013; 382: 209–222. 23680352 10.1016/S0140-6736(13)60844-2

[4] Isenbarger D, Prospective study of the incidence of diarrhoea and prevalence of bacterial pathogens in a cohort of Vietnamese children along the Red River. Epidemiology and Infection 2001; 127: 229–236. 11693500 10.1017/s0950268801005933 PMC2869742

[5] Bodhidatta L, Case-control study of diarrheal disease etiology in a remote rural area in Western Thailand. American Journal of Tropical Medicine and Hygiene 2010; 83: 1106–1109. 21036846 10.4269/ajtmh.2010.10-0367 PMC2963978

[6] World Health Organization. WHO estimates of the global burden of foodborne diseases. Geneva, Switzerland, 2015.

[7] World Health Organization. The global view of campylobacteriosis. Geneva, Switzerland, 2013.

[8] Janssen R, Host-pathogen interactions in *Campylobacter* infections: the host perspective. Clinical Microbiology Reviews 2008; 21: 505–518. 18625685 10.1128/CMR.00055-07 PMC2493085

[9] Humphrey T, O'Brien S, Madsen M. Campylobacters as zoonotic pathogens: a food production perspective. International Journal of Food Microbiology 2007; 117: 237–257. 17368847 10.1016/j.ijfoodmicro.2007.01.006

[10] Coker A, Human campylobacteriosis in developing countries. Emerging Infectious Diseases 2002; 8: 237–244. 11927019 10.3201/eid0803.010233 PMC2732465

[11] Havelaar A, Immunity to *Campylobacter*: its role in risk assessment and epidemiology. Critical Reviews in Microbiology 2009; 35: 1–22. 19514906 10.1080/10408410802636017

[12] Domingues AR, Source attribution of human campylobacteriosis using a meta-analysis of case-control studies of sporadic infections. Epidemiology and Infection 2012; 140: 970–981. 22214729 10.1017/S0950268811002676

[13] European Food Safety Authority (EFSA) and European Centre for Disease Prevention and Control (ECDC). The European Union summary report on trends and sources of zoonoses, zoonotic agents and food-borne outbreaks in 2014. EFSA Journal 2015; 13: 191 pp. 10.2903/j.efsa.2018.5500 PMC7009540 32625785

[14] Mughini-Gras L, Campylobacteriosis in returning travellers and potential secondary transmission of exotic strains. Epidemiology and Infection 2013; 142: 1277–1288. 23962634 10.1017/S0950268813002069 PMC9151200

[15] Hassan KE, The impact of household hygiene on the risk of bacterial diarrhea among Egyptian children in rural areas, 2004–2007. Journal of Infection in Developing Countries 2014; 8: 1541–1551. 25500652 10.3855/jidc.4539

[16] Rao M, Pathogenicity and convalescent excretion of *Campylobacter* in rural Egyptian children. American Journal of Epidemiology 2001; 154: 166–173. 11447051 10.1093/aje/154.2.166

[17] Lengerh A, Prevalence, associated risk factors and antimicrobial susceptibility pattern of *Campylobacter* species among under five diarrheic children at Gondar University Hospital, Northwest Ethiopia. BMC Pediatrics 2013; 13: 1–9. 23694714 10.1186/1471-2431-13-82 PMC3663702

[18] Young JR, Improving smallholder farmer biosecurity in the Mekong Region through change management. Transboundary and Emerging Diseases 2013; 62: 491–504. 26302253 10.1111/tbed.12181

[19] Nampanya S, Improvement in smallholder farmer knowledge of cattle production, health and biosecurity if southern Cambodia between 2008 and 2010. Transboundary and Emerging Diseases 2011; 59: 117–127. 21791034 10.1111/j.1865-1682.2011.01247.x

[20] World Health Organization. Neonatal and child health country profile (http://www.who.int/maternal_child_adolescent/epidemiology/profiles/neonatal_child/khm.pdf). Accessed 21 December 2015.

[21] Darapheak C, Consumption of animal source foods and dietary diversity reduce stunting in children in Cambodia. International Archives of Medicine 2013; 6: 29. 23866682 10.1186/1755-7682-6-29 PMC3720190

[22] Lay KS, Prevalence, numbers and antimicrobial susceptibilities of *Salmonella* serovars and *Campylobacter* spp. in retail poultry in Phnom Penh, Cambodia. Journal of Veterinary Medical Science 2011; 73: 325–329. 21060246 10.1292/jvms.10-0373

[23] Carrique-Mas JJ, An epidemiological investigation of *Campylobacter* in pig and poultry farms in the Mekong delta of Vietnam. Epidemiology and Infection 2014; 142: 1425–1436. 24067502 10.1017/S0950268813002410 PMC4045178

[24] Osbjer K, Household practices related to disease transmission between animals and humans in rural Cambodia. BMC Public Health 2015; 15: 476. 25952633 10.1186/s12889-015-1811-5 PMC4427931

[25] Osbjer K, Detection of *Campylobacter* in human and animal field samples in Cambodia. APMIS 2016; 124: 508–515. 26991032 10.1111/apm.12531

[26] Thrusfield M. Veterinary Epidemiology, 3rd edn. Oxford: Blackwell Science, 2007 pp. 610.

[27] Lindblom G-B, *Campylobacter jejuni/coli* and enterotoxigenic *Eschericia coli* (ETEC) in faeces from children and adults in Tanzania. Scandinavian Journal of Infectious Diseases 1995; 27: 589–593. 8685639 10.3109/00365549509047073

[28] Mason J, *Campylobacter* infection in children in Malawi is common and is frequently associated with enteric virus co-infections. PLoS ONE 2013; 8: e59663. 23555739 10.1371/journal.pone.0059663 PMC3608717

[29] Kassa T, Gebre-Selassie S, Asrat D. Antimicrobial susceptibility patterns of thermotolerant *Campylobacter* strains isolated from food animals in Ethiopia. Veterinary Microbiology 2007; 119: 82–87. 17000061 10.1016/j.vetmic.2006.08.011

[30] Dohoo IR, Veterinary Epidemiologic Research, 2nd edn. Charlottetown: AVC Inc., 2003, pp. 865.

[31] Otte MJ, Gumm ID. Intra-cluster correlation coefficients of 20 infections calculated from the results of cluster-sample surveys. Preventive Veterinary Medicine 1997; 31: 147–150. 9234433 10.1016/s0167-5877(96)01108-7

[32] Denis M, Development of a m-PCR assay for simultaneous identification of *Campylobacter jejuni* and *C. coli*. Letters in Applied Microbiology 1999; 29; 406–410. 10664985 10.1046/j.1472-765x.1999.00658.x

[33] Gonzalez I, Specific identification of the enteropathogens *Campylobacter jejuni* and *Campylobacter coli* by using a PCR test based on the ceuE gene encoding a putative virulence determinant. Journal of Clinical Microbiology 1997; 35: 759–763. 9041429 10.1128/jcm.35.3.759-763.1997 PMC229667

[34] O'Connell AA, Multilevel Modeling of Educational Data. Charlotte: Information Age Publishing Inc., 2008, pp. 199–242.

[35] Vigre H, Intra-unit correlations in seroconversion to *Actinobacillus pleuropneumoniae* and *Mycoplasma hyopneumoniae* at different levels in Danish multi-site pig production facilities. Preventive Veterinary Medicine 2004; 63: 9–28. 15099713 10.1016/j.prevetmed.2004.02.002

[36] Baqui AH, Methodological issues in diarrhoeal disease epidemiology: definition of diarrhoeal episodes. International Journal of Epidemiology 1991; 4: 1057–1063. 10.1093/ije/20.4.1057 1800404

[37] Goldman N, Vaughan B, Pebley AR. The use of calendars to measure child illness in health interview surveys. International Journal of Epidemiology 1998; 27: 505–512. 9698144 10.1093/ije/27.3.505

[38] Randremanana R, Case-control study of the etiology of infant diarrheal disease in 14 districts in Madagascar. PLoS ONE 2012; 7: e44533. 23028555 10.1371/journal.pone.0044533 PMC3444445

[39] da Silva Quetz J, *Campylobacter jejuni* and *Campylobacter coli* in children from communities in Northeastern Brazil: molecular detection and relation to nutritional status. Diagnostic Microbiology and Infectious Disease 2010; 67: 220–227. 20542202 10.1016/j.diagmicrobio.2010.02.025 PMC2886016

[40] Lee G, Symptomatic and asymptomatic *Campylobacter* infections associated with reduced growth in Peruvian children. PLoS Neglected Tropical Diseases 2013; 7: e2036. 23383356 10.1371/journal.pntd.0002036 PMC3561130

[41] Biran A, Comparing the performance of indicators of hand-washing practices in rural Indian households. Tropical Medicine and International Health 2008; 13: 278–285. 18304276 10.1111/j.1365-3156.2007.02001.x

[42] Evans MR, Ribeiro CD, Salmon RL. Hazards of healthy living: bottled water and salad vegetables as risk factors for *Campylobacter* infection. Emerging Infectious Diseases 2003; 9: 1219–1225. 14609455 10.3201/eid0910.020823 PMC3033096

[43] Boer ED, Hahné M. Cross-contamination with *Campylobacter jejuni* and *Salmonella* spp. from raw chicken products during food preparation. Journal of Food Protection 1990; 53: 1067–1068. 31018267 10.4315/0362-028X-53.12.1067

